# Integrating physical activity into the primary school curriculum: rationale and study protocol for the “Thinking while Moving in English” cluster randomized controlled trial

**DOI:** 10.1186/s12889-019-6635-2

**Published:** 2019-04-04

**Authors:** Myrto F. Mavilidi, David R. Lubans, Philip J. Morgan, Andrew Miller, Narelle Eather, Frini Karayanidis, Chris Lonsdale, Michael Noetel, Kylie Shaw, Nicholas Riley

**Affiliations:** 1 Priority Research Centre for Physical Activity and Nutrition, University of Newcastle, Callaghan, NSW 2308 Australia; 2 School of Education, Faculty of Education and Arts, University of Newcastle, University Drive, Newcastle, 2308 Australia; 3 School of Psychology, Faculty of Science, University of Newcastle, University Drive, Newcastle, 2308 Australia; 4 Institute for Positive Psychology and Education, Faculty of Health Sciences, Australian Catholic University, Level 9 & 10, 33 Berry Street, North Sydney, 2060 Australia

**Keywords:** Physical activity, Primary schools, English, On-task behavior, Cognitive function, Randomized controlled trial

## Abstract

**Background:**

The current and declining physical activity levels of children is a global concern. Integrating physical activity into the school curriculum may be an effective way not only to improve children’s physical activity levels but also enhance educational outcomes. Given the recent national focus in Australia on improving the literacy levels of children in primary school, and an increasing proportion of time spent on explicitly teaching these skills, integrating physical activity into English could be a viable strategy to improve literacy levels and physical activity at the same time. The aim of this study is to evaluate the impact of the ‘Thinking While Moving in English’ (TWM-E) program on children’s physical activity, on-task behavior in the classroom, academic achievement, and executive function.

**Methods:**

Grade 3–4 children from 10 public schools in New South Wales, Australia will be randomly allocated to intervention (*n* = 5) or control (*n* = 5) groups. All teachers will receive 1-day workshop of registered professional learning and a TWM-E equipment pack (e.g., chalk, lettered bean bags). Intervention schools will be asked to adapt their English lessons to embed movement-based learning in their daily program for three 40-min lessons per week, over a six-week period. The primary outcome is children’s physical activity levels across the school day (measured using accelerometry). Secondary outcomes are children’s on-task behavior during English lessons, academic achievement in English, and executive function. A detailed process evaluation will be undertaken including questionnaires, fidelity checks, and teacher and student interviews.

**Discussion:**

The TWM-E program has the potential to improve primary school children’s physical activity levels, along with academic outcomes (on-task behavior, cognition, and academic achievement), and provide stakeholders with exemplar lessons and guidelines which illustrate how to teach English to children whilst they are moving.

**Trial registration:**

Australian and New Zealand Clinical trial Register ACTRN12618001009202

Date registered: 15/06/2018 retrospectively registered.

**Electronic supplementary material:**

The online version of this article (10.1186/s12889-019-6635-2) contains supplementary material, which is available to authorized users.

## Background

Participation in physical activity in children is in decline, and this is a concern because physical inactivity is associated with a range of chronic illnesses such as obesity, high blood pressure, type II diabetes, and some cancers [1, 2]. Physical activity can also prevent mental health disorders (e.g., anxiety and depression), and enhance psychological health (e.g., well-being) [3, 4]. Additionally, improvements in physical activity and fitness have been shown to increase students’ school engagement, as well as enhance their cognitive and academic performance [5–8].

Despite the numerous benefits of physical activity, very few children and adolescents are sufficiently active [9]. National and international physical activity recommendations suggest that children should spend at least 60 min in daily physical activity to achieve optimal health outcomes [10–13]. However, reports show that only 19% of Australian children and adolescents meet these recommendations [14, 15].

The International Society for Physical Activity and Health (2012) [16] considers schools as one of the best “investments” for promoting physical activity. School-based programs have been shown to increase children’s physical activity levels [17, 18]. A multi-component approach for encouraging physical activity in schools typically involves the delivery of quality physical education, activity before, during, and after the school day, as well as staff, family, and community involvement [17, 19]. However, the authors of two systematic reviews and meta-analyses concluded that the effects of school-based interventions on objectively measured physical activity are minimal [20, 21]. A possible reason could be that physical activity interventions are rarely implemented as intended, with teachers reporting that lack of time as the most common barrier [18, 22].

A recent systematic review proposed a conceptual model describing neurobiological, psychosocial, and behavioral mechanisms that may account for the positive effects of physical activity on academic outcomes in young people [23]. Neuroimaging studies in children have examined the structural and functional brain changes associated with participation in physical activity. For example, the FitKids trial [24] found that children who participated in a 9-month physical activity program improved their performance on cognitive tasks and demonstrated more mature brain activation patterns in the right anterior prefrontal cortex.

A range of psychosocial mechanisms (e.g., motivation, perceptions of novelty, and attitudes toward physical activity) have emerged as potential mediators of the effects of physical activity on academic outcomes. Although there is little experimental evidence, it is plausible that engagement in physically active lessons may increase students’ enjoyment and interest, and this may indirectly enhance learning. Pesce (2012) [25] suggested that goal-directed, cognitively demanding physical activity can activate the same neurons in the brain that are used to control complex cognitive processes. Finally, on-task behavior is a key predictor of academic success [26] and may explain the effects of school-based physical activity on children’s learning and academic performance.

Growing evidence supports the positive effects of: (i) active recess (i.e., providing opportunities for students to be active during recess and lunch breaks), (ii) classroom physical activity breaks (i.e., short activity breaks delivered in the classroom, known also as energiser breaks), and iii) physically active lessons (i.e., lessons that integrate physical activity into other key learning areas), to increase students’ on-task behavior, academic achievement, and physical activity levels [7, 27]. Among these, the physically active academic lessons have been studied the least.

Physically active academic lessons have the potential to increase children’s physical activity levels during the school day without compromising academic time [19, 27]. Concomitantly, embedding movement-based learning into academic instruction can enhance children’s learning performance [28–35]. Physically active environments have also been shown to improve children’s executive functions [36–38]. Importantly, the core executive functions (i.e., inhibition, shifting, and updating [39]) are fundamental for children’s physical, emotional, psychological, and social development and have been linked with school readiness and academic success [40–42].

A previous iteration of the “Thinking While Moving program” in Maths (TWM published as EASY MINDS) aimed at numeracy, and a pilot version of the “Thinking While Moving in English” (TWM-E) produced promising results when physically active academic lesons are integrated during learning: The TWM in Maths program involved a successful randomized controlled trial (RCT), consisting of 3 × 60 min physically active mathematics lessons for six weeks [34]. Compared with the control group, the intervention group showed higher physical activity during the Mathematics lessons, and on-task behavior. The program was rated as providing positive experiences for teachers and students, both in terms of enjoyment and engagement [35]. A recent TWM-E feasibility trial was carried out in a single school for four weeks and all lesson plans were organized and delivered by the research team [43]. Improvements favoring the intervention group were found in on-task behavior and spelling scores in Grade 4 students. Taking into account effective strategies and teachers’ feedback utilized in the previous “Thinking While Moving” programs the next phase will include a cluster RCT of the TWM-E.

The study described in this protocol will extend the TWM in Maths intervention to English. The TWM-E intervention will involve integrating physical activity into English lessons in primary schools. Current curriculum recommendations in New South Wales (NSW), Australia require primary school students to spend 25–35% of a school week in English lessons, 20% in Mathematics and only 6–10% in Personal Development, Health and Physical Education (PDHPE [44]). Despite this strong emphasis on literacy, national and international assessments indicate that about one quarter of Australian students achieve literacy results at or below the minimum standards [45, 46].

There is clearly a strong rationale for reconsidering the design and delivery of literacy programs in Australian primary schools to reinvigorate English lessons with interactive movement-based learning, given the proportion of time children spend on this traditionally sedentary subject. Combining literacy curricula with physical activity may also have positive effects on both physical and cognitive outcomes (e.g., memory, attention, goal-directed behavior).

### Aims and hypotheses

The overall aim of this RCT is to evaluate the effect of integrating physical activity into English lessons on children’s school-based physical activity, on-task behavior, learning and cognition (i.e., executive function).

The specific research questions for this study include:

i) What is the impact of the TWM-E intervention on the primary outcome, physical activity during the school day?
ii) What is the impact of the TWM-E intervention on the secondary outcomes, on-task behavior during English lessons, academic achievement in English and executive function?

It is hypothesized that the TWM-E group will show improvements in all measures compared to the control group.

## Methods/design

### Participants

TWM-E will be evaluated using a two-arm pararell group cluster RCT with an intervention and a wait-list control group. Stage 2 teachers (Grade 3 & 4) from 10 government primary schools of the Hunter Region, NSW, Australia will be invited to participate. Stage 2 includes the first year of the Australian National Assessment Program for Literacy and Numeracy (NAPLAN) testing in Grade 3 and thus it has been selected as the ideal time for teachers to consolidate identified literacy skills through increasing on-task behavior and engagement [47, 48]. Baseline data collection will occur in the school term preceding the intervention delivery (i.e., Term 2, May to June 2018). The intervention delivery will be conducted in Term 3 (i.e., July to September 2018). Post-test data collection will commence midway through Term 3 and will contintue until the end of the term.

Ethics Approval has been sought and obtained from the University of Newcastle, Australia (No: H-2017-0240), and the NSW Department of Education (SERAP No: 2017368). The TWM-E trial is registered with the Australian and New Zealand Clinical Trials registry (ACTRN12618001008213). Schools will be randomly selected from a metropolitan area (within a 60 km radius of the University of Newcastle). School principals will receive an initial invitation letter followed by an email. Principals, teachers, and parents will need to provide written consent forms for each child to participate.

**Fig. 1:**
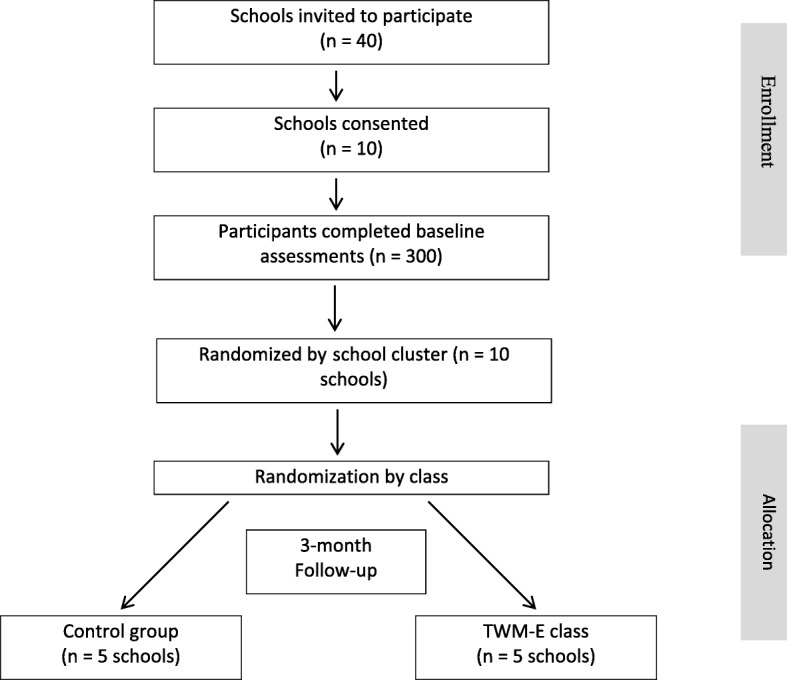
Flow of participants

The design, conduct, and reporting of the TWM-E program will adhere to the Consolidation Standards of Reporting Trials (CONSORT) guidelines and the extension for cluster RCTs [49]. Participating schools will be stratified and pairs of schools will be matched according to their enrolment size and demographics (e.g., literacy program, socio-economic status and location), using the index of community socio-educational advantage (ICSEA). The ICSEA value includes data and information regarding family background (e.g., parental occupation, school/ non-school education achieved) provided to schools directly by families. Following baseline assessments, an independent researcher not involved in the project will use a random number producing algorithm to randomly assign each pair of matched schools to either control or the treatment condition. This method will ensure the same likelihood of allocation into one of the two study arms for all schools. Figure 1 shows the flow of participants. Schools in the waitlist control arm will continue with usual practice, which may include some schools pursuing other physical activity promotion initiatives. No restrictions will be made regarding schools’ participation in such programs, but they will be asked to provide details of their involvement. However, during recruitment, schools that are currently participating in physical activity programs run by the University (e.g., iPLAY [50]) will not be targeted for inclusion.

### Sample size calculation

Power analysis using procedures appropriate for a cluster RCT study design [51] were conducted to determine the sample size required to detect changes in the primary outcome of accelerometer-determined physical activity counts per minute (CPM). Calculations assumed baseline to post-test correlation scores of *r* = 0.30 and were based on 80% power and alpha level 0.05. Based on the reported physical activity effects (i.e., *SD* change = 200 CPM) after six weeks of the TWM in Maths study pilot study, and a conservative intra-class correlation coefficient (ICC = 0.15), a study sample of *N* = 200 with 8 clusters (i.e., schools) of 25 students would provide adequate power to detect a between group difference of 200 CPM across the school day [34, 52].

### Intervention

TWM-E will involve teachers adapting 3 × 40 min English lessons per week over a 6-week period. Movement-based learning will be incorporated during teaching selected curriculum from the NSW K-6 English syllabus. The program delivery will involve the following components: (i) full-day professional learning for teachers, (ii) TWM-E equipment pack, (iii) handbook for teachers with examples on how to incorporate movement-based learning into English lessons, (iv) online and PDF resources with lesson examples developed by the research team, (v) three observations with feedback per school by members of the research team during program implementation, which will also function as a fidelity check, and (vi) weekly newsletter emails providing implementation strategy support (i.e., separate advice and tips per school based on observations by the research team).

Firstly, participating teachers will attend a full-day professional learning workshop conducted at the University and delivered by the research team. The professional learning day will be registered and accredited with the National Educational Standards Authority (NESA [53]; NSW Department of Education, 2017). Teachers will earn professional learning hours towards their teacher accreditation (Highly Accomplished Standards 1.2.3, 2.5.3, 4.1.3, 6.3.3). The purpose of this workshop will be to help teachers familiarize themselves with the process of integrating movement-based learning with appropriate English syllabus content. A training model is useful for the dissemination of key knowledge to be used in the intervention. The mentoring model [54, 55] is underpinned by situated learning theory [56] and contextualizes the theoretical content presented to teachers. A summary of the workshop’s content is presented in Table 1.

**Table 1:** Summary of the professional learning workshop

1. Course rationale	• Presentation of current research findings that identify prevalence and issues around physical activity, cognitive performance and the K-6 English curriculum
2. Evidence for TWM-E	• Findings of the feasibility trial and comparison with the previous Thinking while Moving in Maths research project
3. Becoming a TWM-E advocate	• Demonstration and planning learning sessions: explanatory videos created by the research team focusing on the benefits of school-based physical activity, physical activity and cognition, using physical activity during lessons, why English lessons should become active, how to make it work in every school, and the benefits of the TWM-E approach
4. Implementing TWM-E at schools	• Demonstration of TWM-E activities using existing lessons designed by the research team • Teachers will modify their own lessons based on the TWM- E approach • Presentation of challenges of successfully implementing and advocating TWM-E in their schools (i.e., establishment of clear pedagogical expectations, tasks and strategies that teachers will need to address when they are in the school setting, and design of implementation plans)
5. Reporting on TWM-E in schools (post workshop)	• Teachers will be required to introduce the TWM-E pedagogy into their school community and provide evidence of its delivery and impact • Teachers will also use resources developed and discussed during the workshop to model, advocate and support colleagues as they incorporate TWM-E into their teaching practice

**Fig. 2:**
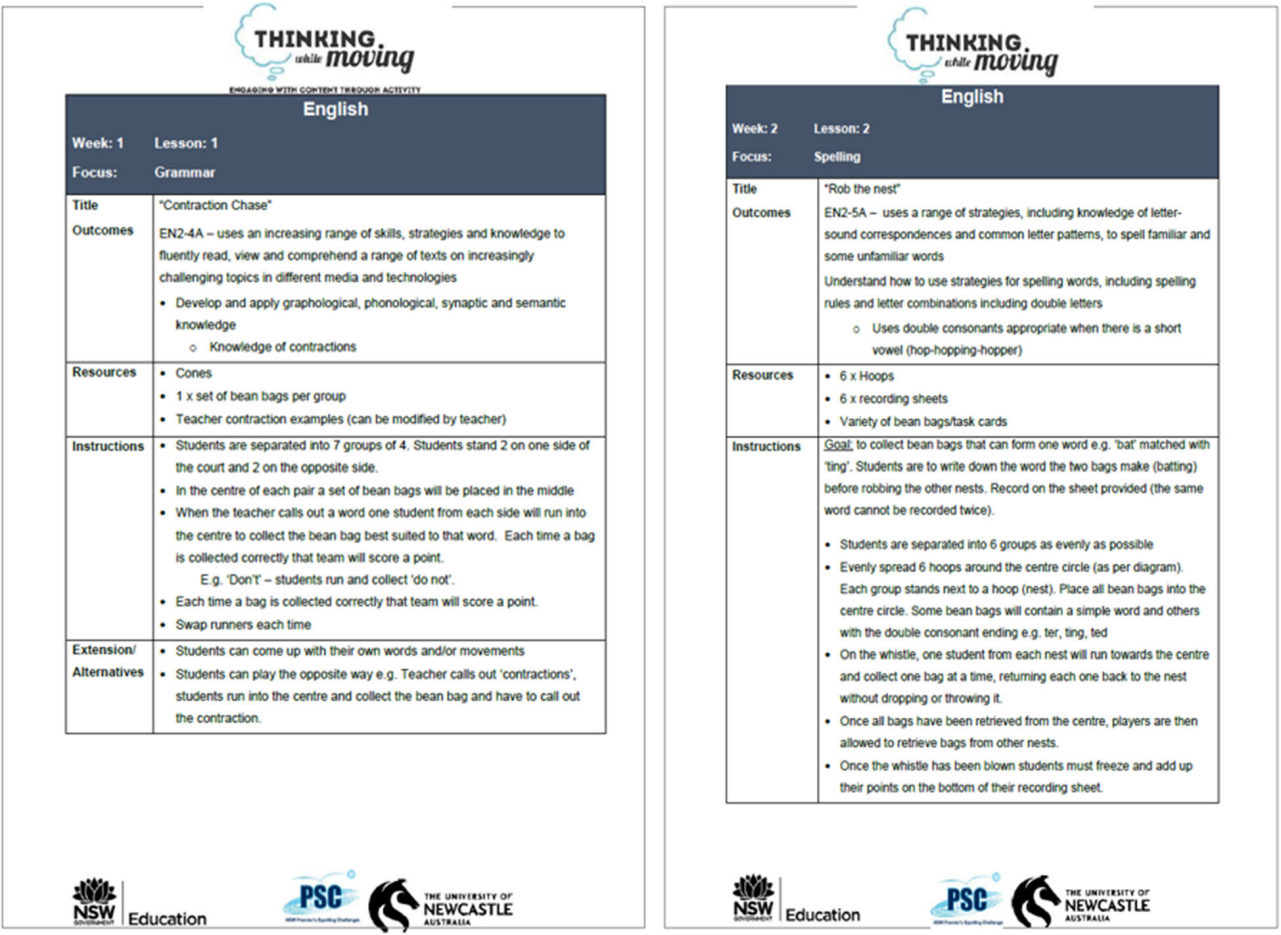
Example of activities and lesson plans

The workshop is designed to equip teachers with the necessary skills and motivation to use physical activity as a teaching approach for the development of literacy skills, focusing on the benefits of physical activity on students’ engagement, academic and cognitive performance. Teachers will be familiarized through demonstration with activities and learning experiences and provided with resources to promote physical activity integration across the primary school English curriculum. A key focus will be maintained on the desired English outcomes from the current syllabus, and more specifically on spelling and grammar, which are content areas that benefit from rehearsal to consolidate learning. Activities will be aligned with NSW English syllabus. Example ideas, activities, and a potential lesson sequence can be found in Fig. 2.

Following the completion of the professional learning workshop, participating schools will receive a teacher-selected TWM-E equipment pack (e.g., chalk, buckets, whiteboards, drill ladders, basketballs, skipping ropes, lettered beanbags, and lettered flexi domes - value $400 AU). Also, teachers will be given access to a website with online modules and movement-based activity examples (via videos) developed by the research team.

The program will be delivered by the regular classroom teacher during the schools’ timetabled English lessons. Teachers will be encouraged to apply their knowledge from the professional development training to prepare and deliver physically active English lessons. All teachers will be emailed a weekly newsletter offering tips and strategies, and will receive three visits during Weeks 1, 3, and 5 of the program. During Weeks 2, 4, and 6, evaluation with feedback will be provided to the teachers by the research team attending while observing the physically active English lessons.

Considerable concerns have been raised from the public health research sector regarding the design and development of interventions that are not feasible to be adopted and implemented in real world settings [57]. A particular emphasis on school-based physical activity has been placed in the state of NSW, Australia, in which government policy requires public schools to offer 150 min of planned physical activity across the school week [58]. TWM-E has been requested by the NSW Department of Education and has been designed to maximize scalability and sustainability. As recommended in the Consolidated Framework for Implementation Research (CFIR), the intervention will target schools, principals, teachers, and students [59]. A summary of the TWM-E components are presented in Table 2. Several implementation strategies have been designed to support the delivery of the TWM-E program including: i) intervention characteristics, ii) outer setting (i.e., educational authorities), iii) inner setting (i.e., schools), iv) characteristics of teachers, and v) implementation process.

**Table 2:** Summary of the TWM-E components

Level	Intervention Component	Dose	Description	Implementation evaluation
Teacher	1) Professional learning workshop	1 × 5 h	1) Course rationale 2) Evidence for TWM-E 3) Becoming a TWM-E advocate 4) Implementing TWM-E at school 5) Reporting on TWM-E in schools	• Teacher post-workshop evaluation questionnaire • Post-program implementation questionnaire (Teacher evaluation of the TWM-E pedagogy)
	2) Session observations	3	The research team will conduct three observations of the TWM-E lessons in weeks 2, 4, and 6 using the evaluation Checklist.	• Evaluations Checklist (Fidelity check) conducted by the research team • Teacher interview questions
	3) Support from the research team	On-going	The research team will be available for the teachers throughout the duration of the presentation. Weekly emails including advice and strategies will be sent during Weeks 1, 3, and 5 of the intervention.	• Post-program implementation questionnaire (Teacher evaluation of the TWM-E pedagogy)
School	4) Dissemination to school staff	1 × 45 min	Teachers will present the TWM-E (e.g., objective and program details) pedagogy during their staff meetings.	• School principals will sign off teachers after the completion of their presentation in MyPL (teachers will receive the full NESA accreditation)
	5) Equipment	Once	Schools will be provided with a TWM-E equipment pack selected by the participating teachers to assist in the delivery of the program (e.g., chalk, buckets, whiteboards, drill ladders, basketballs, skipping ropes, lettered beanbags, and lettered flexi domes - value $AU400).	• Post-program implementation questionnaire (Teacher evaluation of the TWM-E pedagogy)
Student	6) TWM-E lessons	3 / week	TWM-E lessons will be run during curricular English time by the regular school teachers. The TWM-E lessons will last for 40 min.	• Student intervention evaluation • Student interview questions

### Measures

Consenting students will be assessed at two time points using identical measures (baseline and 3-months). Trained research assistants will collect student level assessments, and where possible the same assessors will be used for both time points. Research assistants will be blinded to schools’ allocation to the conditions. More specifically, on-task behavior, followed by the cognitive assessments will be conducted by research staff on the same day at the same time for each time point (i.e., Monday, 9:00 am, baseline and posttest). To avoid participant burden, academic achievement tests will be administered by the classroom teachers on a different day using a standardized data collection protocol given to the teachers by the research team. Students’ demographic information characteristics will be collected at the end of the intervention.

### Physical activity

The primary outcome will be children’s physical activity levels during the school day. Objective measurements of intensity and physical activity levels (i.e., counts per minute) will be obtained using tri-axial wrist-worn accelerometers AX3 (Axivity, York, UK). AX3 accelerometers have been found to be a valid and reliable tool for measuring movements [60] [61].

Accelerometers will be distributed on Monday morning and will be collected Friday afternoon. They will be worn for five consecutive school days from 9:00 am to 3:00 pm. Teachers will be trained to assist their students with placement of the accelerometers. They will distribute and collect the accelerometers daily which will be coded for individual students. Accelerometer data will be downloaded in raw format using OmiGui Software and processed in R software (http://cran.r-project.org/) using the software package GGIR [62]. Data extracted between Monday – Friday (9 am – 3 pm) will be retained for the analysis. Non-wear time will be classified within a 60-min time window if for at least two out of the three axes [63]. Data will be reduced by calculating the average gravity-based acceleration units (g), per 1-s epoch, with daily time spent in moderate-to-vigorous physical activity (MVPA) determined using the sum of epochs averaging above 142 mg [64]. The average minutes spent in MVPA per day and average daily wear time will be computed using data from each participant’s valid days. A valid day will be defined as > 5 school hours on any given day [34], with participants included in the analysis if they wear the monitor for at least 3 days [65].

### Academic outcomes

#### On-task behavior

Time spent on-task during English lessons will be observed using momentary time sampling, reported as a percentage of time, categorized as “on-task” (consisted of “actively engaged” or “passively engaged”), and “off-task” [66]. Active engagement refers to the time a child is actively engaged in an academic activity (e.g., reading, writing, or performing the designated task), rather than passive engagement (i.e., looking at an appropriate target (e.g., the teacher) but not actively engaged in the activity). Off-task behavior is related to behavior not associated with the task (e.g., off-task motor behavior, walking around the class, off-task verbal behavior, chatting, or off-task passive behavior, staring in the class [34, 67]. This observational tool has been adapted from the “Behavior Observation of students in schools” and the “Applied Behavior Analysis for Teachers” [66, 68]. Twelve students (6 boys, 6 girls) will be observed during their English lessons (e.g., 9:00–11:00 am) in 15 s intervals on a rotational basis over a 30-min period in the allocated English time slot. Two lesson observations per school at each time point will be included. Two trained research assistants will observe simultaneously, after receiving a 2-h training focusing on identifying and classifying behavior into the appropriate categories, and a practice trial.

#### Academic achievement

The standardized test “Progressive Achievement Test (PAT)” will be administered in the classroom under 30-min of exam conditions, based on the Australian Curriculum for Education Research (ACER) recommendations [69]. This battery includes different assessments for written spelling (30 items) grammar and punctuation (35 items).

#### Executive function

Cognitive assessments will be measured using the National Institute of Health (NIH) Toolbox Assessment of Neurological and Behavioral Function for 7–17 years (www.nihtoolbox.org), installed on iPad devices [70]. This is a validated instrument for children between 3 and 15 year-old [71] and includes measures of attention, cognitive control, episodic and working memory, language, and processing speed.

Two measures of executive function (i.e., inhibition and cognitive control) will be selected: The Eriksen Flanker task examines the ability to selectively attend and respond to a target stimulus, while resisting interference input that competes with the target. Participants respond with their left or their right hand according to the direction of the central arrow of a multi-arrow display. The central arrow is flanked by arrows that are either congruent (i.e., point in the same direction as the central arrow, ➔ ➔ ➔ ➔ ➔) or incongruent (i.e., point in the other direction, 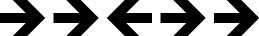). Congruent trials elicit faster and more accurate responses that incongruent trials [72, 73]. Four practice trials are presented prior to the assessment [74]. Twenty trials are conducted for ages > 8. The test takes approximately 3–5 min to administer.

The Dimensional Change Sort Test measures two dimensions of cognitive control: concept formation (i.e., the ability to identify and respond to a specific task-set) and cognitive flexibility (i.e., the ability to use feedback to switch between different task-sets). Participants are presented with stimuli that can be classified according to two dimensions (e.g., shape and colour). They are required to sort the stimuli using one dimension (e.g., trucks vs. cars) irrespective of colour. After a certain number of correct trials, they are required to change rules and sort the stimuli using the other dimension (e.g., red vs. blue pictures). Students are given three practice trials which are directly followed by the examination [74]. This test takes approximately 4–6 min to administer.

In both cognitive measures, scoring will be based on a combination of accuracy and reaction time [74]. Accuracy and reaction time are calculated via a 2-vector scoring method: each of these “vector” values range between 0 and 5, and the computed score that combines the vector scores ranges from 0 to 10. If participants’ accuracy levels are ≥80%, the final “total” computed score is equal to the accuracy score. If participants’ accuracy levels reach < 80%, the accuracy and reaction time score are combined [74].

The uncorrected standard score will be calculated for both cognitive measures, using a standard score metric (normative mean = 100, *SD* = 15). Participants’ overall level of cognitive functioning will be compared with the entire NIH toolbox U.S. representative normative sample, regardless of any demographic characteristics (e.g., age, gender). High scores indicate higher performance [74].

### Process evaluation

The feasibility of the TWM-E program will be assessed using a range of measures including adherence, as well as teachers’ and students’ satisfaction. The following aspects of intervention implementation will be evaluated [75]: (i) *Fidelity and quality:* we will conduct three lesson observations per teacher during the program period using a semi-structured observation checklist (Table 3), (ii) *Responsiveness:* teachers’ evaluation of the TWM-E workshop, resources and their own lessons, and (iii) *Adaptation:* teachers’ modifications of TWM-E program resources (Additional file 1).

**Table 3:** Evaluation checklist (Fidelity check)

Date:	Start Time:	Finish Time:				
English content						
Physical Activity						
(*Please circle and provide comments)*		*(1 = Not at all true* to *5 = Very true)*				
English concepts	i) Key English concepts were reinforced throughout the movement-based activity	1	2	3	4	5
	ii) Movement aided and promoted learning	1	2	3	4	5
	iii) Students were given feedback to support their English knowledge and understanding	1	2	3	4	5
Activity levels	i) Transitions were managed smoothly	1	2	3	4	5
	ii) Students assisted in the set-up and collection of equipment	1	2	3	4	5
	iii) Equipment used promoted physical activity	1	2	3	4	5
Engagement	i) Students were engaged by the activities taught	1	2	3	4	5
	ii) Students remained on-task throughout the lesson	1	2	3	4	5
	iii) Students enjoyed the movement-based English lesson	1	2	3	4	5
	Comments:					

Adherence (how many teachers completed program components): During the intervention period, teachers will be provided with weekly support, including advice and strategies by the research team during Weeks 1, 3, and 5 of the intervention. The lessons will also be observed during Weeks 2, 4 and 6, focusing on physically active English concepts (*n* = 3), activity levels (*n* = 3), and students’ engagement (*n* = 3). During the lesson observations, components of the workshop regarding English content and physical activity (i.e., engagement and activity levels) that will be adopted and adapted to the classes will be assessed through an evaluation checklist (see Table 3).

Teacher satisfaction: After the completion of the professional learning day, teachers will respond to the questionnaire assessing their perceptions on the skills acquired from the training, satisfaction and quality of the training, and their confidence to deliver physically active English lessons (Additional file 1). Teachers’ responses will be recorded answering on a 5-point Likert scale ranging from 1, ‘strongly disagree’, to 5, ‘strongly agree’. They will also have the opportunity through an open-ended response to provide the research team suggestions for improving the workshop and/or the program.

Teacher and student satisfaction: At the end of the 6-week intervention, students will complete an anonymous evaluation questionnaire of their perceptions of physically active English lessons both on enjoyment and perceived learning (Additional file 1). Responses will be ranked on a 5-point Likert scale ranging from 1, ‘strongly disagree’, to 5, ‘strongly agree’. Finally, at the end of the program, teachers and students will be invited to participate in semi-structured interviews (teachers) and focus groups (students) regarding their perceptions of the program (i.e., nature and quality of English lessons prior to and after the program, whether the TWM-E approach influenced their perceptions related to English and physical activity promotion; See Additional file 1). These measures will be adapted from the previously developed evaluation tools from the TWM program [76].

### Statistical analyses

Primary and secondary outcomes will be analyzed through linear mixed models, as they are robust to the biases of missing data and provide appropriate balance of Type 1 and Type 2 errors [77, 78]. Taking into account the hierarchical structure of the data in educational research (e.g., students nested within classes and schools), multilevel modelling analyses can be linked to several predictor variables at the individual level (e.g., students) and at a group levels (e.g., classrooms, schools [79, 80]). In this study, the models will be specified to adjust for the clustered nature of the data (i.e., class level was included as a random intercept) including all randomized participants in the analysis. Previous studies have shown that school-level clustering is negligible after accounting for clustering at the class level [81]. Analyses will be conducted using SPSS (version 22) and alpha levels will be set at *p* > 0.05. Data will be analyzed according to intention-to-treat principles. Qualitative data from teachers and students semi-structured interviews will be processed using a standard general inductive approach to qualitative analysis [82, 83]. Data will be transcribed verbatim, and then will be coded using thematic analysis.

## Discussion

The primary aim of this study is to assess the impact of a curriculum-based physical activity intervention program, integrating physical activity into English lessons, on children’s physical activity levels. The secondary aims are to examine the impact of the program on children’s on-task behavior, academic achievement in English and executive function. The program will be delivered by classroom teachers, after receiving 1-day professional learning workshop. Previous intervention programs that integrate physical activity during learning have been shown to increase physical activity levels and are perceived as the preferred instructional method by children [28, 30–32, 34, 35, 43].

In addition, these programs have found positive effects on children’s academic achievement (e.g., language [26, 29, 33, 84, 85]). Using task-relevant movements which can be translated into academic concepts is suggested to be an effective way of learning due to the mental connection of the physical with the cognitive task [32]. Likewise, physical activity interventions have been proven to foster executive function skills in children [36, 86, 87]. Specifically, cognitively enriched physical activity programs, including chronic physical activity with cognitive training, have found improvements in primary school children’s shifting performance [38, 88], inhibition and updating [86]. Inhibition and shifting were also improved in adolescents through acute effects of exercise (i.e., single bouts [36, 89]).

Lastly, the current physical activity intervention emphasizes the significant role of the teacher on intervention outcomes [90]. The provision of high-quality professional learning development to teachers is necessary for promoting fundamental changes to occur in children’s physical activity [91, 92]. Giving flexibility to teachers to design and develop their learning lessons based on a movement-based curriculum will possibly increase sustainability of the program and allow teachers to integrate it across other grades and curriculum areas.

The cluster RCT design is a significant strength of this study, including quantitative and qualitative measures to explore the program feasibility. In addition, the program was designed based on previous successful studies [34, 43]. The protocol of the study includes detailed process evaluations taking into account students’ and teachers’ point of views. Identifying strengths, but also challenges and barriers, will ensure that the physical activity programs combined with academic instruction are addressing students’ and teachers’ needs with the perspective of a longer-term implementation even after the end of the intervention duration. Such programs, including stealth interventions, are considered the most effective way to foster physical activity [93].

Concluding, the current study may provide time-constrained solution for schools focusing on the dual goal of increasing physical activity and academic achievement. The suggested instructional approach of TWM-E allows educators to decide how to implement these activities in the classroom based on shared quidelines. This flexible implementation increases the likelihood that the program will be adopted by other teachers, overcoming existing barriers, and promoting the dissemination of this study in primary schools across Australia. The partnership with the NSW Department of Education School Sport Unit increases the sustainability of this program in the long-run.

## Additional file

Additional file 1: **Table S1.** Teacher post-program evaluation of TWM-E pedagogy. **Table S2.**Teacher post-workshop evaluation questionnaire. **Table S3.**Student intervention evaluation. **Table S4.**Teacher & student interview questions. (DOCX 26 kb)

## Abbreviations

CONSORT: Consolidation standards of reporting trials
CPM: Counts per minute
ICSEA: Index of community socio-educational advantage
MVPA: Moderate-to-vigorous physical activity
NAPLAN: National assessment program for literacy and numeracy
NIH: National Institute of Health
NSW: New South Wales
PAT: Progressive achievement test
PDHPE: Personal development, health, and physical education
RCT: Randomized controlled trial
SPSS: Statistical package for social sciences
TWM: Thinking while Moving
TWM-E: Thinking while Moving in English

## References

[1] Janssen I, LeBlanc AG. Systematic review of the health benefits of physical activity and fitness in school-aged children and youth. Int J Behav Nutr Phys Act. 2010;7(40). 10.1186/479-5868-7-40. 10.1186/1479-5868-7-40 PMC2885312 20459784

[2] Poitras VJ, Gray CE, Borghese MM, Carson V, Chaput J-P, Janssen I (2016). Systematic review of the relationships between objectively measured physical activity and health indicators in school-aged children and youth. Appl Physiol Nutr Metab.

[3] Biddle SJH, Asare M (2011). Physical activity and mental health in children and adolescents: a review of reviews. Brit J Sports Med.

[4] Babic M, Morgan PJ, Plotnikoff RC, Lonsdale C, White RL, Lubans DR (2014). Physical activity and physical self-concept in youth: systematic review and meta-analysis. Sports Med.

[5] Álvarez-Bueno C, Pesce C, Cavero-Redondo I, Sánchez-López M, Garrido-Miguel M, Martínez-Vizcaíno V (2017). Academic achievement and physical activity: a meta-analysis. Pediatrics..

[6] Khan NA, Hillman CH (2014). The relation of childhood physical activity and aerobic fitness to brain function and cognition: a review. Pediatr Exerc Sci.

[7] Owen KB, Parker PD, Van Zanden B, MacMillan F, Astell-Burt T, Lonsdale C (2016). Physical activity and school engagement in youth: a systematic review and meta-analysis. Educ Psych.

[8] Santana CCA, Azevedo LB, Cattuzzo MT, Hill JO, Andrade LP, Prado WL (2017). Physical fitness and academic performance in youth: a systematic review. Scand J Med Sci Sports.

[9] Hallal PC, Bo Andersen L, Bull FC, Guthold R, Haskell W, Ekelund U (2012). Global physical activity levels: surveillance progress, pitfalls, and prospects. Lancet..

[10] Australian Government Department of Health. Australia’s physical activity and sedentary behaviour guidelines 2017 [cited Commonwealth of Australia]. Retrieved March 18, 2019 from: http://www.health.gov.au/internet/main/publishing.nsf/content/health-pubhlth-strateg-phys-act-guidelines#npa05.

[11] Canadian Society for Exercise Physiology. Canadian 24-hour movement guidelines Ontario, Canada. 2017. Retrieved March 18, 2019 from: http://csepguidelines.ca/.

[12] Centers for Disease Control and Prevention. Cancer prevention and control. Current physical activity guidelines. Atlanta, USA2016. Retrieved March 18, 2019 from: https://www.cdc.gov/cancer/dcpc/prevention/policies_practices/physical_activity/guidelines.htm.

[13] World Health Organization. Physical activity Geneva, Switzerland. 2018. Retrieved March 18, 2019 from: https://www.who.int/news-room/fact-sheets/detail/physical-activity.

[14] Crawford D (2009). The future of sport in Australia.

[15] Hardy L, Mihrshahi S, Drayton B, Bauman A. NSW Schools Physical Activity and Nutrition Survey (SPANS). Full report. 2016 NSW Department of Health; 2017.

[16] Global Advocacy for Physical Activity (GAPA) the Advocacy Council of the International Society for Physical Activity and Health, NCD prevention: Investments that work for physical activity. Br J Sports Med. 2012;46:709–12. 10.1136/bjsm.2012.091485 22869788

[17] Centers for Disease Control and Prevention. Comprehensive school physical activity programs: A guide for schools. Atlanta: US Department of Health & Human Services. USA.gov; 2013.

[18] van Sluijs EMF, McMinn AM, Griffin SJ (2007). Effectiveness of interventions to promote physical activity in children and adolescents: systematic review of controlled trials. BMJ..

[19] Hills AP, Dengel DR, Lubans DR (2015). Supporting public health priorities: recommendations for physical education and physical activity promotion in schools. Progress Card Dis.

[20] Metcalf B, Henley W, Wilkin T (2012). Effectiveness of intervention on physical activity of children: systematic review and meta-analysis of controlled trials with objectively measured outcomes. BMJ..

[21] Borde R, Smith JS, Sutherland R, Nathan N, Lubans DR (2017). Methodological considerations and impact of school-based interventions on objectively measured physical activity in adolescents: a systematic review and meta-analysis. Obes Rev.

[22] Naylor P, Nettlefold L, Race D, Hoy C, Ashe MC, Higgins JW (2015). Implementation of school based physical activity interventions: a systematic review. Prev Med.

[23] Lubans DR, Richards J, Hillman CH, Faulkner G, Beauchamp MR, Nilsson M (2016). Physical activity for cognitive and mental health in youth: a systematic review of mechanisms. Pediatrics..

[24] Chaddock-Heyman L, Erickson KI, Voss MW, Knecht AM, Pontifex MB, Castelli DM (2013). The effects of physical activity on functional MRI activation associated with cognitive control in children: a randomized controlled intervention. Front Hum Neurosci.

[25] Pesce C (2012). Shifting the focus from quantitative to qualitative exercise characteristics in exercise and cognition research. J Sport Exercise Psy.

[26] Donnelly JE, Lambourne K (2011). Classroom-based physical activity, cognition, and academic achievement. Prev Med.

[27] Watson A, Timperio A, Brown H, Best K, Hesketh KD (2017). Effect of classroom-based physical activity interventions on academic and physical activity outcomes: a systematic review and meta-analysis. Int J Behav Nutr Phys Act.

[28] Mavilidi MF, Okely A, Chandler P, Domazet SL, Paas F. Immediate and delayed effects of integrating physical activity into preschool children’s learning of numeracy skills. J Exp Child Psychol. 2018;166:502–19. 10.1016/j.jecp.2017.09.009 29096234

[29] Mavilidi MF, Okely AD, Chandler P, Cliff DP, Paas F. Effects of integrated physical exercises and gestures on preschool children’s foreign language vocabulary learning. Educ Psychol Rev. 2015;27(3):413–26.

[30] Mavilidi MF, Okely AD, Chandler P, Paas F. Effects of integrating physical activities into a science lesson on preschool children's learning and enjoyment. Mind Brain Educ. 2017;31(3):281–90.

[31] Mavilidi MF, Okely AD, Chandler P, Paas F (2016). Infusing physical activities into the classroom: effects on preschool children’s geography learning. Appl Cogn Psychol.

[32] Mavilidi MF, Ruiter M, Schmidt M, Okely AD, Loyens S, Chandler P, et al. A narrative review of school-based physical activity for enhancing cognition and learning: the importance of relevancy and integration. Front Psychol. 2018;9. 10.3389/fpsyg.2018.02079 PMC6234858 30464752

[33] Toumpaniari K, Loyens S, Mavilidi MF, Paas F. Preschool children’s foreign language vocabulary learning by embodying words through physical activity and gesturing. Educ Psychol Rev. 2015;27(3):445–56.

[34] Riley N, Lubans DR, Holmes K, Morgan PJ (2016). Findings from the EASY Minds cluster randomized controlled trial: evaluation of a physical activity integration program for mathematics in primary schools. J Phys Act Health.

[35] Riley N, Lubans DR, Holmes K, Gore J, Hansen V, Morgan PJ (2017). Movement-based mathematics: enjoyment and engagement without compromising learning through the EASY Minds program. Eurasia J Math Sci Tech Educ.

[36] Hillman CH, Pontifex MB, Raine LB, Castelli DM, Hall EE, Kramer AF (2009). The effect of acute treadmill walking on cognitive control and academic achievement in preadolescent children. Neuroscience..

[37] Egger F, Conzelmann A, Schmidt M (2018). The effect of acute cognitively engaging physical activity breaks on children's executive functions: too much of a good thing?. Psychol Sport Exerc.

[38] Schmidt M, Jäger K, Egger F, Roebers CM, Conzelmann A (2015). Cognitively engaging chronic physical activity, but not aerobic exercise, affects executive functions in primary school children: a group-randomized controlled trial. J Sport Exerc Psychol.

[39] Miyake A, Friedman NP, Emerson MJ, Witzki AH, Howerter A, Wager TD (2000). The unity and diversity of executive functions and their contributions to complex “frontal lobe” tasks: a latent variable analysis. Cogn Psychol.

[40] Diamond A, Lee K (2011). Interventions shown to aid executive function development in children 4 to 12 years old. Interventions shown to aid executive function development in children 4 to 12 years old.

[41] Diamond A (2013). Executive functions. Annu Rev Psychol.

[42] Schmidt M, Egger F, Benzing V, Jäger K, Conzelmann A, Roebers CM (2017). Disentangling the relationship between children’s motor ability, executive function and academic achievement. PLoS One.

[43] Mavilidi MF, Lubans DR, Eather N, Morgan PJ, Riley N. Preliminary efficacy and feasibility of “thinking while moving in English”: a program with physical activity integrated into primary school English lessons. Children. 2018;5(8):109. 10.3390/children5080109 PMC6111322 30103471

[44] NSW Education Standards Authority. Stage Statements and time allocation kindergarten to year 6 2018. Retrieved March 18, 2019 from: http://educationstandards.nsw.edu.au/wps/portal/nesa/k-10/understanding-the-curriculum/curriculum-syllabuses-NSW.

[45] NSW Board of Studies (2014). Parents’ guide to the NSW primary syllabuses Sydney, Australia.

[46] Hempenstall K. Read about it : scientific evidence for effective teaching. Jennifer B, editor: Centre for Independent Studies (Australia); 2016.

[47] NSW Department of Education. Literacy and Numeracy Strategy 2017–20. 2017 Retrieved March 18, 2019 from: http://www.dec.nsw.gov.au/about-the-department/our-reforms/literacy-and-numeracy-strategy.

[48] Thomson S, Hillman K, Wernert N, Schmid M, Buckley S, Munene A. Monitoring Australian year 4 student achievement internationally: TIMSS and PIRLS 2011. Melbourne: Australian Council for Educational Research (ACER); 2012.

[49] Moher D, Hopewell S, Schulz KF, Montori V, Gøtzsche PC, Devereaux PJ, et al. CONSORT 2010 explanation and elaboration: updated guidelines for reporting parallel group randomised trials. BMJ. 2010;340. 10.1136/bmj.c869. 10.1136/bmj.c869 PMC2844943 20332511

[50] Lonsdale C, Sanders T, Cohen KE, Parker P, Noetel M, Hartwig T (2016). Scaling-up an efficacious school-based physical activity intervention: study protocol for the ‘internet-based professional learning to help teachers support activity in youth’ (iPLAY) cluster randomized controlled trial and scale-up implementation evaluation. BMC Public Health.

[51] Donner A, Klar N (2000). Design and analysis of cluster randomization trials in health research.

[52] Riley N, Lubans DR, Holmes K, Morgan PJ. Rationale and study protocol of the EASY Minds (Encouraging Activity to Stimulate Young Minds) program: cluster randomized controlled trial of a primary school-based physical activity integration program for mathematics. Rationale and study protocol of the EASY Minds (Encouraging Activity to Stimulate Young Minds) program: cluster randomized controlled trial of a primary school-based physical activity integration program for mathematics. 2014;14. 10.1186/1471-2458-14-816 PMC4137101 25103358

[53] NSW Department of Education. National Education Standards Authority Accreditation (NESA) 2017. Retrieved March 18, 2019 from: https://education.nsw.gov.au/about-us/jobs-and-opportunities/school-careers/teachers/nesa-accreditation.

[54] Kennedy A (2005). Models of continuing professional development: a framework for analysis. Models of continuing professional development: A framework for analysis.

[55] Rhodes C, Beneicke S (2003). Professional development support for poorly performing teachers: challenges and opportunities for school managers in addressing teacher learning needs. J Inservice Educ.

[56] Lave J, Wenger E (1991). Situated learning: legitimate peripheral participation.

[57] Milat AJ, King L, Bauman AE, Redman S (2013). The concept of scalability: increasing the scale and potential adoption of health promotion interventions into policy and practice. Health Promot Int.

[58] NSW Department of Education. Policy library. Sport and physical activity policy 2018. Retrieved March 18, 2019 from: https://education.nsw.gov.au/policy-library/policies/sport-and-physical-activity-policy.

[59] Damschroder LJ, Aron DC, Keith RE, Kirsh SR, Alexander JA, Lowery JC. Fostering implementation of health services research findings into practice: a consolidated framework for advancing implementation science. Implement Sci. 2009;4(50). 10.1186/1748-5908-4-50 PMC2736161 19664226

[60] Feng Y, Wong CK, Janeja V, Kuber R, Mentis HM (2017). Comparison of tri-axial accelerometers step-count accuracy in slow walking conditions. Gait & Posture.

[61] Hickey A, Del Din S, Rochester L, Godfrey A (2017). Detecting free-living steps and walking bouts: validating an algorithm for macro gait analysis. Physiol Meas.

[62] van Hees VT, Gorzelniak L, Dean León EC, Eder M, Pias M, Taherian S (2013). Separating movement and gravity components in an acceleration signal and implications for the assessment of human daily physical activity. PLoS One.

[63] Sabia S, van Hees VT, Shipley MJ, Trenell MI, Hagger-Johnson G, Elbaz A (2014). Association between questionnaire- and accelerometer-assessed physical activity: the role of sociodemographic factors. Am J Epidemiol.

[64] Hildebrand M, Hees VT, Hansen BH, Ekelund U. Age-group comparability of raw accelerometer output from wrist-and hip-worn monitors. Med Sci Sports Exerc. 2014:46. 10.1249/MSS.0000000000000289 24887173

[65] Cain KL, Sallis JF, Conway TL, Van Dyck D, Calhoon L (2013). Using accelerometers in youth physical activity studies: a review of methods. J Phys Act Health.

[66] Alberto PA, Troutman AC. Applied behavior analysis for teachers. London, UK: Pearson Education; 2003.

[67] Mahar MT, Murphy SK, Rowe DA, Golden J, Shields AT, Raedeke TD (2006). Effects of a classroom-based program on physical activity and on-task behavior. Med Sci Sports Exerc.

[68] Shapiro ES, Cole CL. Behavior change in the classroom: self-management interventions. New York, NY, USA: Guilford Press; 1994.

[69] Stephanou A, Anderson P, Urbach D. PAT-R progressive achievement tests in Reading: comprehension, vocabulary and spelling. Camberwell, Victoria: Australian Council for Educational Research; 2008.

[70] Health Measures. NIH Toolbox. 2018. Retrieved March 18, 2019 from: http://www.healthmeasures.net/explore-measurement-systems/nih-toolbox.

[71] Lockhart KL, Keil FC (2018). The proper realms of medicines and their alternatives: what count as cures?. Monogr Soc Res Child Dev.

[72] Eriksen CW, Schultz DW (1979). Information processing in visual search: a continuous flow conception and experimental results. Percept Psychophys.

[73] Hillman CH, Snook EM, Jerome GJ (2003). Acute cardiovascular exercise and executive control function. Int J Psychophysiol.

[74] Slotkin J, Nowinski C, Hays R, Beaumont J, Griffith J, Magasi S, et al. NIH Toolbox scoring and interpretation guide [Measurement instrument]. National Institutes of Health and Northwestern University; 2012. Retrieved March 18, 2019 from: https://www.epicrehab.com/epic/documents/crc/crc-201307-nih-toolbox-scoring-and-interpretation-manual%209-27-12.pdf.

[75] Durlak JA, DuPre EP. Implementation matters: a review of research on the influence of implementation on program outcomes and the factors affecting implementation. Am J Community Psychol. 2008;41. 10.1007/s10464-008-9165-0 18322790

[76] Riley N, Lubans DR, Holmes K, Morgan PJ. Rationale and study protocol of the EASY Minds (encouraging activity to stimulate young Minds) program: cluster randomized controlled trial of a primary school-based physical activity integration program for mathematics. BMC Pub Health. 2014;14(819). 10.1186/471-2458-14-816. 10.1186/1471-2458-14-816 PMC4137101 25103358

[77] Krull JL, MacKinnon DP (2001). Multilevel modeling of individual and group level mediated effects. Multivariate Behav Res.

[78] Mallinckrodt CH, Watkin JG, Molenberghs G, Carroll RJ, Lilly E (2004). Choice of the primary analysis in longitudinal clinical trials. Pharm Stat.

[79] Snijders T, Bosker R (1999). Multilevel modeling: an introduction to basic and advanced multilevel modeling.

[80] Raudenbush SW, Bryk A. Hierarchical linear models: applications and data analysis methods. 2nd ed. Thousands Oaks, CA: Sage; 2002.

[81] Rosenkranz RR, Lubans DR, Peralta LR, Bennie A, Sanders T, Lonsdale C (2012). A cluster-randomized controlled trial of strategies to increase adolescents’ physical activity and motivation during physical education lessons: the motivating active learning in physical Education (MALP) trial. BMC Pub Health..

[82] Braun V, Clarke V. Using thematic analysis in psychology. Qual Res Psychol. 2006;3.

[83] Thomas DR (2006). A general inductive approach for analyzing qualitative evaluation data. Am J Eval.

[84] Kibbe DL, Hackett J, Hurley M, McFarland A, Schubert KG, Schultz A, et al. Ten years of TAKE 10!((R)): integrating physical activity with academic concepts in elementary school classrooms. Prev Med. 2011;52. 10.1016/j.ypmed.2011.01.025 21281670

[85] Mullender-Wijnsma MJ, Hartman E, de Greeff JW, Doolaard S, Bosker RJ, Visscher C. Physically active math and language lessons improve academic achievement: A cluster randomized controlled trial. Pediatrics. 2016;137(3). 10.1542/peds.2015-2743 26912206

[86] Crova C, Struzzolino I, Marchetti R, Masci I, Vannozzi G, Forte R (2014). Cognitively challenging physical activity benefits executive function in overweight children. J Sport Sci.

[87] Stroth S, Kubesch S, Dieterle K, Ruchsow M, Heim R, Kiefer M (2009). Physical fitness, but not acute exercise modulates event-related potential indices for executive control in healthy adolescents. Brain Res.

[88] Crova C, Marchetti R, Struzzolino I, Masci I, Vannozzi G, Forte R (2013). Searching for cognitively optimal challenge point in physical activity for children with typical and atypical motor development. Ment Health Phys Act.

[89] Tomporowski PD, Davis CL, Lambourne K, Gregoski M, Tkacz J (2008). Task switching in overweight children: effects of acute exercise and age. J Sport Exerc Psychol..

[90] Cothran DJ, Kulinna PH, Garn AC (2010). Classroom teachers and physical activity integration. Teaching Teach Educ..

[91] Avalos B (2011). Teacher professional development in teaching and teacher education over ten years. Teaching Teach Educ.

[92] Lander N, Eather N, Morgan PJ, Salmon J, Barnett LM (2017). Characteristics of teacher training in school-based physical education interventions to improve fundamental movement skills and/or physical activity: a systematic review. Sports Med.

[93] Robinson TN, Dubé L, Bechara A, Dagher A, Drewnowski A, Lebel J, James P (2010). Chapter 25 - Stealth Interventions for Obesity Prevention and Control: Motivating Behavior Change. Obesity Prevention.

