# Supplementary material for: Integrating physical activity into the primary school curriculum: rationale and study protocol for the “Thinking while Moving in English” cluster randomized controlled trial
Source: BMC Public Health. 2019 Apr 4;19:379. doi: 10.1186/s12889-019-6635-2 (PMC6449912; doi:10.1186/s12889-019-6635-2)
Supplement: Supplementary file 1 — Table S1. Teacher post-program evaluation of TWM-E pedagogy. Table S2.Teacher post-workshop evaluation questionnaire. Table S3.Student intervention evaluation. Table S4.Teacher & student interview questions. (DOCX 26 kb) [file 12889_2019_6635_MOESM1_ESM.docx]

**Additional file 1**

*Table S1. Teacher post-program evaluation of TWM-E pedagogy.*

**Professional learning workshop and pedagogy resources**

| 1. The workshop prepared me to implement TWM-E pedagogy. |
| --- |
|  |
| 2. I would recommend the workshop to other teachers. |
| 3. The workshop would be just as useful if delivered online. |
| 4. The videos were useful and helped me to design lessons using TWM-E principles. |
| 5. The demonstration of lesson ideas gave me confidence to implement TWM-E in my school. |

**Implementation of the pedagogy**

| 6. The resources at my school were sufficient to deliver the TWM-E program. |
| --- |
| 7. I found it easy to implement the TWM-E pedagogy at my school. |
| 8. There were unexpected barriers to implementing the TWM-E pedagogy. |
| 9. Other teachers at my school were supportive of the TWM-E pedagogy. |
| 10. I enjoyed implementing TWM-E in my school. |

**Impact and benefits for students**

| 11. Involvement in TWM-E improved students’ overall English achievement. |
| --- |
| 12. Involvement in TWM-E improved students’ concentration and on-task behavior in class. |
| 13. Overall, students enjoyed learning when TWM-E principles were applied. |
| 14. The TWM-E presentation was well received by my colleagues. |
| 15. My school plans to continue using the TWM-E pedagogy. |

16. In my school, I implemented the TWM-E principles (please tick):

- Only during English lessons
- In English and in other lessons

*Table S2.Teacher post-workshop evaluation questionnaire.*

| **Overall:**   1. The TWM-E workshop was enjoyable. |
| --- |
| b The TWM-E workshop improved my knowledge about PA integration. |
| c. The TWM-E workshop provided me with useful information and skills that may improve my teaching. |
| **Introduction, academic evidence:** |
| 1. It provided a strong rationale for the TWM-E program. |
| 1. The theoretical background helped outline the importance of the program. |
| **Delivering and evaluating** |
| 1. Working alongside peers was a positive learning experience for me. |
| 1. Peer evaluation was a valuable tool to acquire. |
| 1. I now feel more confident to teach a movement-based English lesson. |
| 1. The resource book and lessons will be extremely valuable. |

*Table S3.* *Student intervention evaluation.*

| 1. TWM-E lessons are enjoyable. |
| --- |
| 2. I like TWM-E lessons that are combined with physical activity. |
| 3. I find it easier to concentrate after being physically active. |
| 4. My teacher enjoys teaching active English lessons. |
| 5. I look forward to TWM-E lessons. |
| 6. TWM-E lessons help me be more active. |
| 7. I like learning outside the classroom.  8. Which TWM-E activities did you find the most enjoyable?  9. Were there any parts of TWM-E that you did not enjoy?  10. Do you have any additional comments about the TWM-E that you think they might be useful for the future? |

Note: Questions 1-7 were answered on a 5-point Likert scale. Questions 8-10 were open-ended.

*Table S4.Teacher & student interview questions*.

Teacher semi-structured interviews

| 1. Can you tell me about your experiences with the TWM-E program? 2. Did you enjoy teaching an active English session as opposed to a classroom-based lesson? 3. What were the major challenges to you as a teacher of active English sessions? 4. Do you think your students enjoyed the lessons? Why/ why not? Have you noticed any changes with your students? 5. What do you think were the benefits of an active English class for you and your students? 6. How well do you they think the students understood the English content in the physically active lessons? Can you give me a specific example? 7. Do you think the physical activity aspect of the lesson contributed to greater engagement in the lesson compared to how that same English content would usually be taught?   Suggestions for improvement   1. Is there anything that could be changed to improve TWM-E? 2. What was the best thing about being involved in TWM-E? 3. Do you have anything else to say about TWM-E? 4. Are you likely to continue with this approach after the study? 5. Have you disseminated this information to other staff members? 6. How did students contribute to the lesson activities? Was this successful?     Student semi-structured interviews (focus groups)   1. Can you tell me what the TWM-E program is all about? 2. How would you describe your English classes before the TWM-E program? Did you enjoy this? 3. Did you enjoy the TWM-E lessons? Why? Can you give me an example? 4. Did you enjoy moving in the classroom? Did this make the English’s activities more interesting? 5. What kinds of activities did you enjoy doing in the TWM-E program? 6. What kinds of activities didn’t you enjoy doing in the TWM-E program? 7. Can you tell me if being active in English class helped you learn? Why/why not? If so can you give me an example? Was it fun and enjoyable   Suggestions for improvement   1. What was the best thing about being involved in TWM-E? 2. Is there anything that could be changed to make TWM-E better? 3. Do you have anything else to say about TWM-E? |
| --- |
